# A numerical simulation of neural fields on curved geometries

**DOI:** 10.1007/s10827-018-0697-5

**Published:** 2018-10-11

**Authors:** R. Martin, D. J. Chappell, N. Chuzhanova, J. J. Crofts

**Affiliations:** School of Science and Technology, Nottingham Trent University, Nottingham, UK

**Keywords:** Neural fields, Collocation method, Non-flat geometries, Geodesics

## Abstract

Despite the highly convoluted nature of the human brain, neural field models typically treat the cortex as a planar two-dimensional sheet of ne;urons. Here, we present an approach for solving neural field equations on surfaces more akin to the cortical geometries typically obtained from neuroimaging data. Our approach involves solving the integral form of the partial integro-differential equation directly using collocation techniques alongside efficient numerical procedures for determining geodesic distances between neural units. To illustrate our methods, we study localised activity patterns in a two-dimensional neural field equation posed on a periodic square domain, the curved surface of a torus, and the cortical surface of a rat brain, the latter of which is constructed using neuroimaging data. Our results are twofold: Firstly, we find that collocation techniques are able to replicate solutions obtained using more standard Fourier based methods on a flat, periodic domain, independent of the underlying mesh. This result is particularly significant given the highly irregular nature of the type of meshes derived from modern neuroimaging data. And secondly, by deploying efficient numerical schemes to compute geodesics, our approach is not only capable of modelling macroscopic pattern formation on realistic cortical geometries, but can also be extended to include cortical architectures of more physiological relevance. Importantly, such an approach provides a means by which to investigate the influence of cortical geometry upon the nucleation and propagation of spatially localised neural activity and beyond. It thus promises to provide model-based insights into disorders like epilepsy, or spreading depression, as well as healthy cognitive processes like working memory or attention.

**Electronic supplementary material:**

The online version of this article (10.1007/s10827-018-0697-5) contains supplementary material, which is available to authorized users.

## Introduction

The nervous system consists of approximately 10^11^ neurons and 10^14^ connections all embedded within a highly constrained anatomical space. To better understand such a complex multi-scale system, neural models are deployed that use a range of mathematical and computational techniques to explain/predict function and behaviour of the brain at a range of different scales (Amari 1977; Jirsa and Haken 1996). One such approach, the foundations of which were laid in the 1970s by Wilson and Cowan (1972), and Amari (1977), is neural field theory, which employs a continuum approach to model the activity of large populations of neurons in the cortex. These techniques are of great interest, not only from a mathematical point-of-view, but also from an experimental neuroscience point-of-view, since they can replicate many of the dynamic patterns of brain activity that are observed using modern neuroimaging methodologies (Bojak et al. 2011; Coombes 2010; Sanz-Leon et al. 2015).

Neural field models (NFM) are built from neural masses and typically take the form of a nonlinear partial integro-differential equation of the form

1 $$ \frac{\partial}{\partial t} u(\mathbf{x},t) = -u(\mathbf{x},t) + {\int}_{{\Omega}}{w(\mathbf{x},\mathbf{x}^{\prime})S(u(\mathbf{x}^{\prime},t))\mathrm{d}{\Omega}{(\mathbf{x}^{\prime})}}. $$

**Fig. 1:**
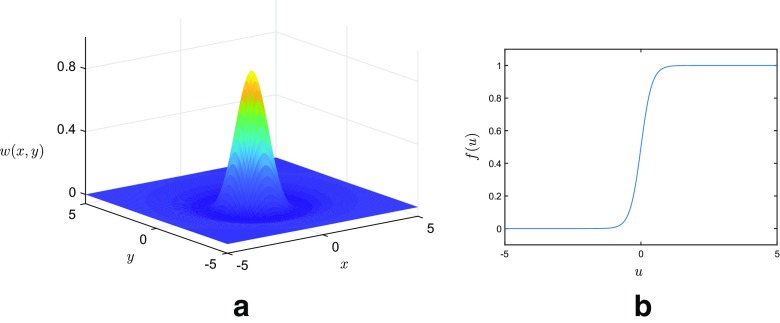
**a** The Mexican hat connectivity function **b** Sigmoidal firing rate function

Here *u*(**x**, *t*) describes the average activity of the neuronal population at position **x** ∈Ω at time *t* ∈ [0,*T*], while the nonlinear function *S* represents the mean firing rate, and typically takes the form of a sigmoid function (see Fig. 1b), although other popular choices include the Heaviside function and piecewise linear functions (Coombes 2010). The connectivity kernel, *w*, describes how neurons positioned at **x** and $\mathbf {x}^{\prime }$ interact, and commonly takes the form of a mexican-hat function (see Fig. 1a). Importantly, the mexican-hat connectivity function admits translational invariance and so depends only on the distance between any two points, that is $w(\mathbf {x},\mathbf {x}^{\prime })\equiv w(d(\mathbf {x},\mathbf {x}^{\prime }))$, where *d* is a suitably defined metric on Ω. Note that the choice of metric reflects the underlying geometry and so, for example, if ${\Omega } = \mathbb {R}^{2}$, then *d* denotes Euclidean distance; however, in this work we also consider NFMs in the case were Ω = *M* is a closed, two-dimensional surface, in which case *d* represents the geodesic distance between points, as defined by the induced metric on *M*.

Techniques for solving NFMs such as (1) typically involve either transforming the problem to an equivalent differential equation (Laing and Troy 2003; Laing 2013), which can be investigated using a mixture of well established analytical and/or numerical methods, or, via direct numerical simulation of the integral form of (1) using fast Fourier transforms (FFTs) to efficiently solve the convolution integral (Rankin et al. 2014). The above approaches, however, rely either on special choices for the integral kernel (*e.g.* kernels, *w*, whose Fourier transform is a rational function) or are restricted to uniform, periodic domains. In the present paper, we combine collocation techniques with efficient numerical algorithms for computing geodesics in order to solve NFMs such as (1) on closed, two-dimensional surfaces, thus opening up the possibility of extending these methods to analyse cortical structures.

Recent studies suggest that myriad neurological conditions are accompanied by alterations in cortical folding (Zhang et al. 2009; Wobrock et al. 2010; White and Hilgetag 2011) and so it is of great importance to investigate the relation between neuroanatomy and brain dynamics. However, whilst a number of recent studies have investigated the relation between surface morphology and large-scale brain connectivity of both grey and white matter (O’Dea et al. 2013; Henderson and Robinson 2014; Lo et al. 2015), and, to a lesser extent the effect of curvature on reaction-diffusion models of neural activity (see, for example, Kneer et al. (2014), Kroos et al. (2016) and references therein), the role that cortical geometry plays in non-local models of brain activity, such as the NFM given by (1), is less well-studied. Some progress in this direction was recently made by Visser et al. (2017), who used a time-delayed NFM to investigate the behaviour of both standing and travelling wave solutions on a sphere; however, their approach is restricted to geometries for which a closed-form for the distance function, *d*, exists. Motivated by the above, in this paper we put forward a novel technique for solving NFMs on general, closed two-dimensional surfaces, the overarching aim of which is to improve understanding of the effects that cortical features, such as fissures and sulci, have on activation spreading dynamics in both healthy and diseased brains.

The paper is organised as follows. In Section 2 we introduce the NFM studied in this work, before going on to discuss the method of collocation as applied to integro-differential equations such as that in (1). We finish Section 2 by giving a brief overview of the Mitchell, Mount and Papadimitriou (MPP) algorithm (Mitchell et al. 1987) for computing geodesics on polygonal surfaces, which allows us to consider NFMs on non-flat domains. In Section 3, we present the results of applying our numerical techniques to solve a NFM on a flat, periodic square domain, the closed surface of a torus and the cortical surface of a rat brain. In the first two cases, we perform a comparative analysis against more standard techniques, deploying either Fourier based methods and/or the trapezoidal rule to compute the integral in (1), and investigate the dependence of our results on the underlying mesh. We then consider solutions of our NFM on the folded structure of the rat brain, which allows us to highlight the extent to which cortical geometry influences solutions of our NFM. We finish in Section 4 by giving an overview of the work as well as explaining its possible implications, before outlining a number of possibilities for future studies.

## Methods

### Governing neural field model

We consider a two-dimensional neural field model of the type studied in Laing (2014):

2 $$ \begin{array}{ll} \frac{\partial u(\mathbf{x},t)}{\partial t} &= A{\int}_{\Omega} w(\mathbf{x},\mathbf{x}^{\prime})S(u(\mathbf{x}^{\prime},t)-h)\mathrm{d}{\Omega}{(\mathbf{x}^{\prime})} \\ & -u(\mathbf{x},t) -a(\mathbf{x},t),\\ \tau \frac{\partial a(\mathbf{x},t)}{\partial t}&=Bu(\mathbf{x},t)-a(\mathbf{x},t). \end{array} $$

The above includes an additional recovery variable *a* which acts to repolarize *u* via negative feedback, while the parameters *A*, *B*, *h* and *τ* are related to the sensitivities and time-scale of the problem (Laing 2014). As mentioned above, the integral kernel $w(\mathbf {x},\mathbf {x}^{\prime })$ describes interactions between neighbouring neurons. We take the following functional form for *w* in our work

3 $$ w(\mathbf{x},\mathbf{x}^{\prime}) = e^{-d(\mathbf{x},\mathbf{x}^{\prime})^{2}} - 0.17e^{-0.2d(\mathbf{x},\mathbf{x}^{\prime})^{2}}, $$

which is a mexican-hat type function, and we choose *d* to be a suitably defined metric; see Fig. 1a for the case in which ${\Omega }=\mathbb {R}^{2}$. In addition, we take *S* be a sigmoid of the form

$$S(u) = \frac{1}{1+e^{-\beta u}}, $$

which converts population activity to firing frequency at a rate governed by the steepness parameter *β*. In all of our experiments the model parameters are chosen as in Laing (2014), that is

$$A = 2, B = 0.4, h = 0.8, \tau = 3 \text{ and } \beta = 5.$$

### Collocation method

Collocation is an example of a projection method that approximates an infinite dimensional problem, such as (2), by a finite dimensional one via a suitably defined projection operator $\mathcal {P}_{n}$. In what follows we provide brief details of the method with piecewise linear interpolation as applied to (2) (further details can be found in the book by Atkinson 1997).

Consider the triangulation $\mathcal {T}_{n} = \{\triangle _{1},...,\triangle _{n}\}$ of the domain Ω and suppose that on each triangle △_*k*_ we employ a piecewise linear approximation of the unknown functions *u*(**x**, *t*) and *a*(**x**, *t*). In this case the projection operator takes the form

$$\begin{array}{@{}rcl@{}} \mathcal{P}_{n} u(\mathbf{x},t) &=& u_{n}(\mathbf{x},t)\\ &=&\sum\limits_{j = 1}^{3}u(\mathbf{v}_{k,j},t)l_{j}(\mathbf{x}), \quad \mathbf{x} \in \triangle_{k}, k = 1,\ldots,n. \end{array} $$

Here, **v**_*k*, *j*_ denotes the coordinates of the *j*^th^ interpolation point of the *k*^th^ triangle △_*k*_, while *l*_*j*_ denotes the linear Lagrange basis functions (Atkinson 1997). Note that a similar projection can be applied to *a*(**x**, *t*).

The above allows us to formulate the following approximation to (2):

4 $$ \begin{array}{ll} \frac{\partial u_{n}(\mathbf{x},t)}{\partial t} &= A\mathcal{P}_{n} \left\lbrace {\int}_{\Omega} w(\mathbf{x},\mathbf{x}^{\prime})S(u(\mathbf{x}^{\prime}, t)-h)\mathrm{d}{\Omega}{(\mathbf{x}^{\prime})} \right\rbrace \\ &-u_{n}(\mathbf{x},t)-a_{n}(\mathbf{x},t), \\ \tau \frac{\partial a_{n}(\mathbf{x},t)}{\partial t} & = Bu_{n}(\mathbf{x},t)-a_{n}(\mathbf{x},t). \end{array} $$

Assuming this expression holds exactly at the node values, $\mathbf {v}_{1}, \mathbf {v}_{2}, \ldots , \mathbf {v}_{n_{v}}$, where *n*_*v*_ refers collectively to a global numbering of the node points **v**_*k*, *j*_, we obtain a collocation scheme for (2).

To make the above collocation scheme more tractable we perform the integration in (4) by applying a quadrature rule over each triangle and summing the result. More specifically, we employ the transformation $T_{k} :\sigma \rightarrow \triangle _{k}$, given by

$$\mathbf{x}^{\prime}=T_{k}(r,s)=(1-r-s)\mathbf{v}_{k,1}+s\mathbf{v}_{k,2}+r\mathbf{v}_{k,3}, $$

which maps the unit simplex *σ* on to each triangle △_*k*_. This enables us to integrate an arbitrary function, *g* say, over the triangle △_*k*_ as follows

$${\int}_{\triangle_{k}} g(\mathbf{x}^{\prime})\mathrm{d}\mathbf{x}^{\prime} = 2\text{Area}(\triangle_{k}){\int}_{\sigma}g(T_{k}(r,s))\mathrm{d}r\mathrm{d}s. $$

Here, the factor of 2Area(Δ_*k*_) appears due to the Jacobian determinant of the transformation *T*_*k*_, which is given by

$$\left|\det\left( DT_{k}\right)(r,s)\right| = \left|\det\left( \begin{array}{ll} x_{k,2}-x_{k,1} & x_{k,3}-x_{k,1}\\ y_{k,2}-y_{k,1}&y_{k,3}-y_{k,1} \end{array}\right)\right| = 2\text{Area}({\Delta}_{k}), $$

where **v**_*k*, *i*_ = (*x*_*k*, *i*_,*y*_*k*, *i*_), *i* = 1,2,3, are the vertices of the *k* th triangle, Δ_*k*_ (see Atkinson (1997) for further details).

Substituting the above quadrature expression into (4), and evaluating at the node values **v**_*i*_, *i* = 1,2,…,*n*_*v*_, gives

5 $$\begin{array}{@{}rcl@{}} \frac{\mathrm{d}u_{n}(\mathbf{v}_{i})}{\mathrm{d}t}&=& -u_{n}(\mathbf{v}_{i})-a_{n}(\mathbf{v}_{i}) + 2A\left( \sum\limits_{k = 1}^{n}\text{Area}(\triangle_{k}) \times\right.\\ &&{}{\int}_{\sigma} \left.w(\mathbf{v}_{i}, T_{k}(r,s))S\left( \sum\limits_{j = 1}^{3} u(\mathbf{v}_{k,j})l_{j}(r,s)-h\right) \mathrm{d}r\mathrm{d}s\right),\\ \frac{\mathrm{d}a_{n}(\mathbf{v}_{i})}{\mathrm{d}t}&=&\frac{1}{\tau}\left( Bu_{n}(\mathbf{v}_{i})-a_{n}(\mathbf{v}_{i})\right), \quad\text{ for } i = 1,2,\ldots, n_{v}. \end{array} $$

The above results in a system of 2*n*_*v*_ ordinary differential equations that can be solved to determine approximate solutions to (2). We have suppressed the *t* dependence in (5) for brevity.

### Efficient computation of geodesics

The key difference between solving (2) on a curved surface as opposed to a planar domain (such as $\mathbb {R}^{2}$) is the computation of the distance function, $d(\mathbf {x}, \mathbf {x}^{\prime })$, appearing in (3), which, except in a very small number of cases, must be computed numerically. To compute geodesic distances in our work, we employed the *exact geodesic toolbox* (O’Rourke 1999), which is a MATLAB implementation of the MMP (Mitchell, Mount and Papadimitriou) algorithm (Mitchell et al. 1987). Developed in 1987, the MMP algorithm solves the *discrete geodesic* problem, *i.e.* it finds the shortest path between two points, *s* and *t* say, on an arbitrary polyhedral surface; it does this by simulating the continuous propagation of a wave front of points equidistant from the source point *s* until the target *t* is reached. The method is reminiscent of Dijkstra’s algorithm for computing the minimum distance between vertex pairs on a graph (Dijkstra 1959) and is therefore often referred to as the *continuous Dijkstra* algorithm.

## Numerical results

In this section we present the results of a number of numerical experiments that were undertaken in order to check the validity of the aformentioned techniques. Of particular importance is our ability to reproduce solutions on generic, irregular triangulations, such as those obtained from neuroimaging studies, and so we begin by investigating the effects of mesh regularity on solutions of (2) on a flat, periodic domain, before moving onto look at more general, curved domains.

### Planar domain with periodic boundary conditions

When considering the numerical solution of (2) the main source of error is the approximation of the integral, which for Ω = [−*L*, *L*]^2^, becomes

6 $$ I = {\int}_{-L}^{L}{\int}_{-L}^{L} w((x,y),(x^{\prime},y^{\prime}))S(u(x^{\prime},y^{\prime})-h)\mathrm{d}x^{\prime}\mathrm{d}y^{\prime}. $$

We note that since $d(\mathbf {x},\mathbf {x}^{\prime }) = \sqrt { (x'-x)^2+(y-y^{\prime })^{2}}$ then the integrals in (6) are of convolution type.

**Fig. 2:**
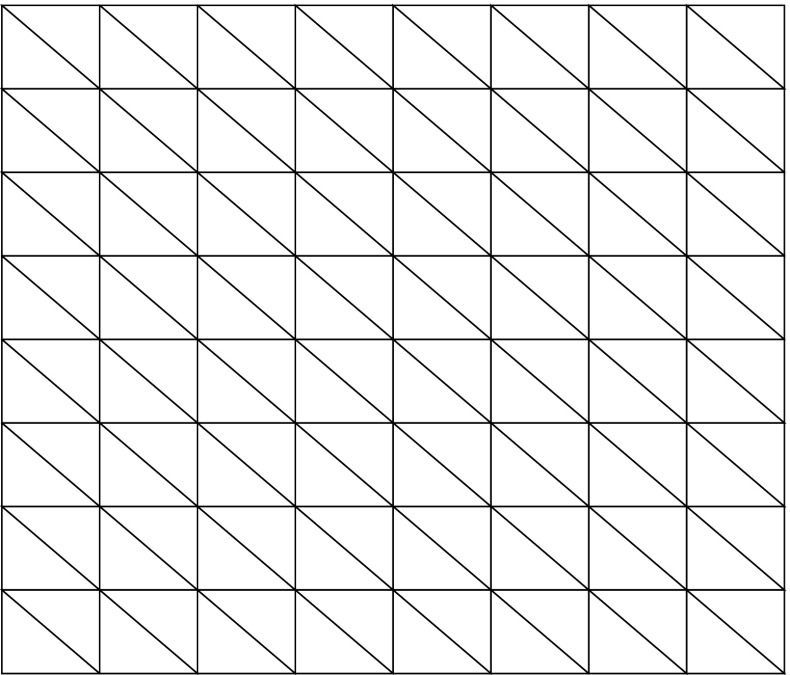
Illustration of a domain that uses Cartesian grid points as triangle vertices

We compared the accuracy of computing the integral in (6) using linear collocation against fast Fourier transform (FFT) techniques together with the convolution theorem, and the trapezoidal method, both of which require a regular spatial discretisation on a Cartesian grid. In order to compare these methods directly to piecewise linear collocation, we employ a triangulation whose vertices correspond to the Cartesian grid points for the other two approaches, as shown in Fig. 2. In our experiments we fixed *L* = 7.5 and set $u(\mathbf {x}^{\prime })= w(0,\mathbf {x}^{\prime })$, *i.e.* the connectivity kernel given in (3), since it is qualitatively similar to the bump solutions admitted by (2). It is worth noting, however, that similar results are obtained for other sufficiently smooth choices of *u* (results not shown). To investigate grid convergence, we considered a sequence of refinements of an initial, regular grid consisting of *N*_0_ = 81 nodes, such that at the *m* th stage of refinement, the number of nodes is given by *N*_*m*_ = (2^*m*^ ⋅ 8 + 1)^2^ for *m* = 1,2,…,7. If we then denote by *I*_*m*_ the numerical approximation of (6) on the grid of size *N*_*m*_, we can approximate the order of convergence of the respective discretisation schemes by considering a log-log plot of the absolute error between consecutive grids, |*I*_*m*+ 1_ − *I*_*m*_|, versus grid size, *N*_*m*+ 1_. Here we consider point-wise convergence and so all results shown are for a representative grid point. Note that we have repeated the analysis for other grid points and observed almost identical behaviour (experiments not shown).

**Fig. 3:**
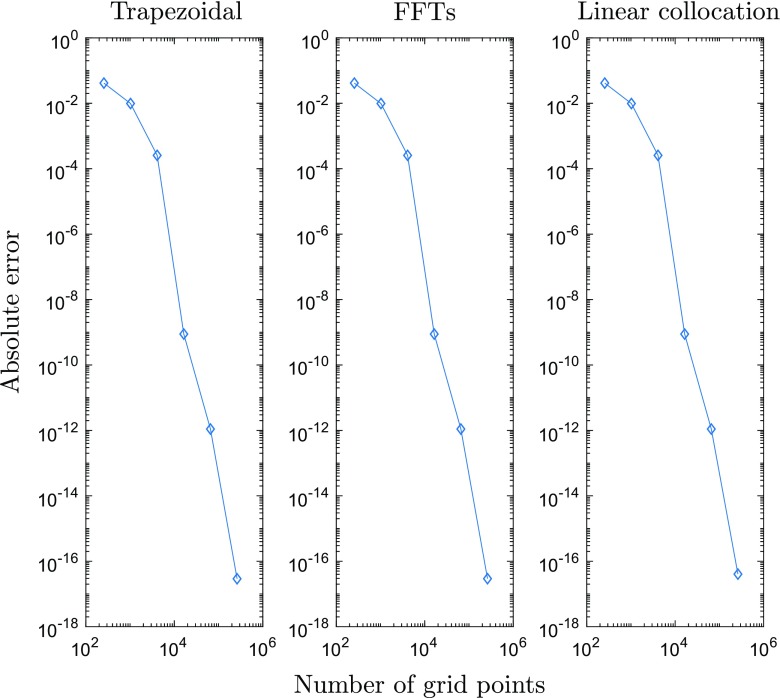
The error |*I*_*m*+ 1_ − *I*_*m*_| plotted against grid size *N*_*m*+ 1_ reveals geometric convergence rates for trapezoidal rule, FFTs and linear collocation when computing the integral in (6)

Our results are displayed in Fig. 3. In particular, we see that both the trapezoidal rule and FFTs display geometric convergence, as expected (see the review by Trefethen and Weideman (2014) for a discussion of the convergence properties of the trapezoidal rule on a periodic domain); however, we find, perhaps somewhat surprisingly, that linear collocation also exhibits the same geometric convergence. To understand the above result, we consider the collocation technique as applied to (6) in more detail below.

Firstly, note that employing linear collocation alongside the three point quadrature rule

$${\int}_{\sigma} G(r,s)\mathrm{d}r\mathrm{d}s= \frac{1}{6}[G(0,0)+G(0,1)+G(1,0)], $$

with *G*(*r*, *s*) = *g*(*T*_*k*_(*r*, *s*)), as defined in Section 2, enables us to construct the following numerical approximation to (6):

7 $$\begin{array}{@{}rcl@{}} \begin{array}{lll}I \approx\sum\limits_{k = 1}^{n}&\frac{\text{Area} \left( \triangle_{k}\right)}{3} \left[\rule{0cm}{0.8cm}\right. w(\mathbf{v}, T_{k}(0,0))S\left( \sum\limits_{j = 1}^{3}u(\mathbf{v}_{k,j})l_{j}(0,0)-h\right)\\&+ w(\mathbf{v}, T_{k}(0,1))S\left( \sum\limits_{j = 1}^{3}u(\mathbf{v}_{k,j})l_{j}(0,1)-h\right)\\ &+ w(\mathbf{v}, T_{k}(1,0))S\left( \sum\limits_{j = 1}^{3}u(\mathbf{v}_{k,j})l_{j}(1,0)-h\right) \left.\rule{0cm}{0.8cm}\right]. \end{array} \end{array} $$

We can further simplify the above by noting that since we are solving on a uniform Cartesian domain, $\text {Area}\left (\triangle _{k}\right )$ = Δ*x*^2^/2 for all triangles, where here, Δ*x* (= Δ*y*) is the local mesh spacing. Substituting this into (7) and evaluating the Lagrange basis functions at the node points gives

$$\begin{array}{@{}rcl@{}} \frac{{\Delta} x^{2}}{6}\sum\limits_{k = 1}^{n} \left[\rule{0cm}{0.8cm}\right. &w(\mathbf{v}, T_{k}(0,0))S(u(\mathbf{v}_{k,1})-h) +\\ &w(\mathbf{v}, T_{k}(0,1))S(u(\mathbf{v}_{k,2})-h)+\\ &w(\mathbf{v}, T_{k}(1,0))S(u(\mathbf{v}_{k,3})-h) \left.\rule{0cm}{0.8cm}\right]. \end{array} $$

Recalling that *T*_*k*_(0,0) denotes the coordinates of the first vertex in △_*k*_, *T*_*k*_(0,1) the second and *T*_*k*_(1,0) the third, we can rewrite the above equation as follows

8 $$\begin{array}{@{}rcl@{}} \begin{array}{ll}\sum\limits_{k = 1}^{n}&\left[\rule{0cm}{0.8cm}\right.w(\mathbf{v}, \mathbf{v}_{k,1})S(u(\mathbf{v}_{k,1})-h) + w(\mathbf{v}, \mathbf{v}_{k,2})S(u(\mathbf{v}_{k,2})-h)\\ &+ w(\mathbf{v}, \mathbf{v}_{k,3})S(u(\mathbf{v}_{k,3})-h)\left.\rule{0cm}{0.8cm}\right]\frac{{\Delta} x^{2}}{6}. \end{array} \end{array} $$

However, since the triangle vertices are simply the Cartesian grid points, (8) is nothing other than the trapezoidal rule for solving (6) on a periodic two-dimensional domain. The factor of 1/6 occurs due to the fact that each node appears six times in the sum in (8). Thus, we have shown that for a regular grid with periodic boundary conditions solving (6) using linear collocation and a quadrature rule based only on the triangle vertices is equivalent to using the trapezoidal rule. This explains the spectral convergence observed in Fig. 3.

**Fig. 4:**
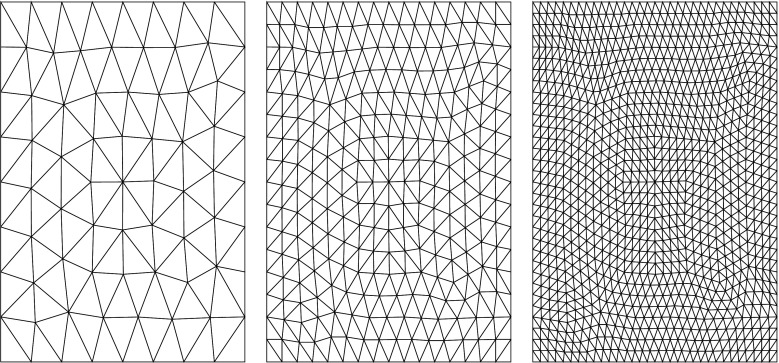
Illustration of the refinement procedure for a general triangulated domain in which the initial mesh is generated using the DistMesh package

**Fig. 5:**
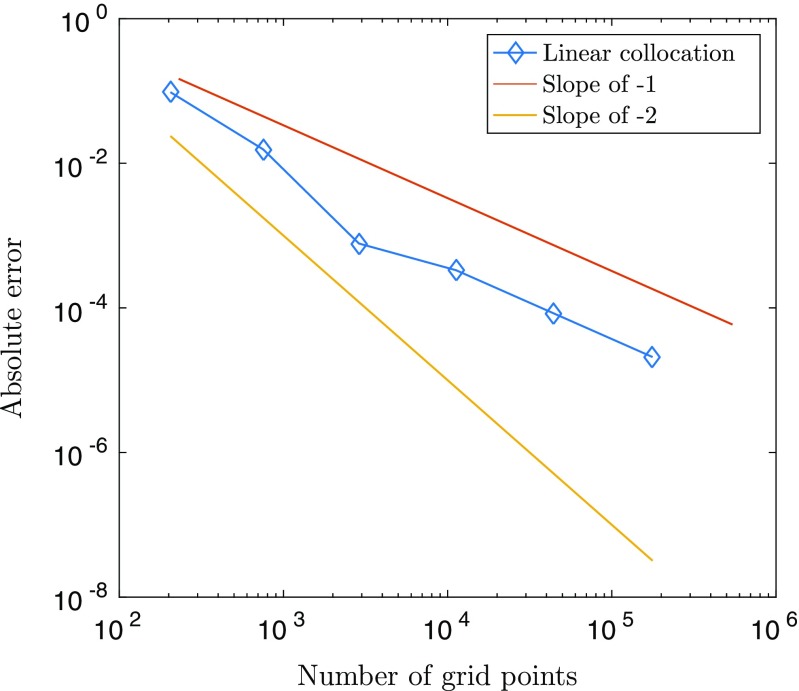
Convergence of linear collocation when computing the integral in (6) on a general triangulation constructed using the DistMesh package. The orange and yellow lines indicate the slopes for first and second order methods, respectively

Next, we considered the effects of mesh regularity on the accuracy of computing the integral in (6). To do this we deployed the DistMesh MATLAB package (Persson and Strang 2004) to generate a general mesh, that is, one in which the triangle vertices do not lie on a Cartesian grid, as in our previous investigations. It is important to note that standard techniques such as those deployed above (*i.e.* trapezoidal and FFT methods) cannot be applied in this more general setting. As before, numerical errors were approximated by comparing the numerical solution of (6) at the same grid point across a range of increasingly fine meshes. More precisely, we constructed an intial, coarse triangulation of the square [−*L*, *L*]^2^ consisting of *N*_0_ = 79 nodes using the DistMesh package, we then proceeded to refine this triangulation by subdividing each triangle into four smaller triangles, as illustrated in Fig. 4. Note that boundary nodes were fixed in all of our experiments in order to implement the periodicity of the problem more easily. Our results are displayed in Fig. 5. In particular, we see that in contrast to our earlier results, the geometric convergence breaks down and we recover linear convergence as expected.

**Fig. 6:**
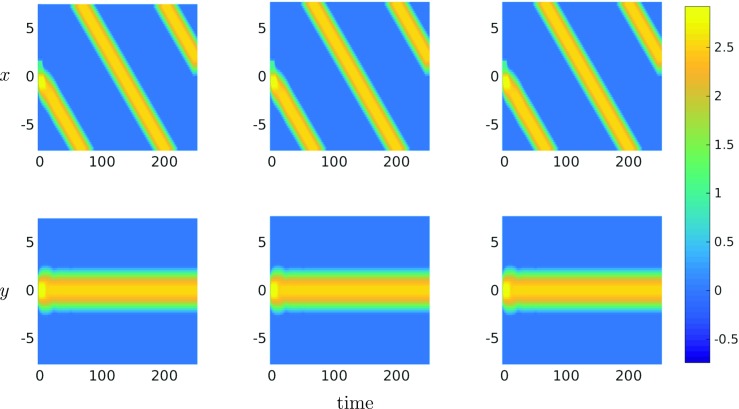
Travelling bump solutions of (2) computed on a triangulation based on a Cartesian grid, using trapezoidal, FFTs and linear collocation, respectively

We solved (2) using the collocation techniques described previously on both regular and irregular meshes. In the case of the Cartesian mesh we also solved using trapezoidal and FFTs, for comparative purposes. In all cases, the neural activation *u* was initially set equal to 1 in a rectangular area centred at the origin, and the recovery variable *a* was set equal to 1.5 in a rectangular area shifted to the right of this initial stimulus, thus determining the propagation of the travelling bump solution from right to left. After spatial discretisation, we integrated the resulting system of ODEs (see (5) in the case of collocation) for *T* = 250 using the built-in MATLAB routine ode45, with absolute and relative tolerances both set to 1e − 6. Figure 6 shows a travelling bump solution centred on the *x*-axis, and moving from right to left, for the trapezoidal, FFT and linear collocation methods, using a regular grid on *n*_*v*_ = 4225 nodes. As expected from our previous analysis, all three methods are in excellent agreement, converging to the same solution up to machine precision.

**Fig. 7:**
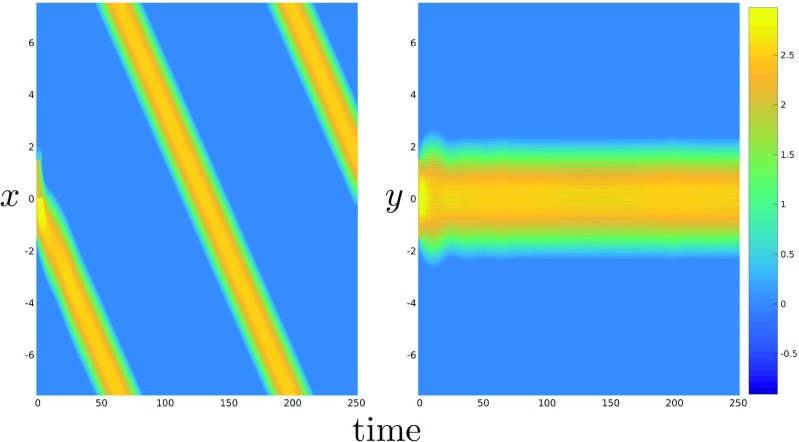
Travelling bump solution of (2) computed on a general DistMesh triangulation

When moving to more general meshes, however, we find that the bump solution tends to drift slightly in the *y*-direction for grids with a similar number of points as the regular ones considered above. Note that we have conducted experiments with varying numbers of spatial grid points and have observed a relationship between the time-step at which the bump solution drifts from *y* = 0 and grid size *n*_*v*_. In particular, Fig. 7 shows a solution computed on a grid consisting of *n*_*v*_ = 13000 nodes for which the aforementioned drift is negligible for the integration times considered here. From our earlier analysis, it is clear that the observed drift is a manifestation of errors due to the linear rate of convergence of the collocation scheme when computing the integral in (6) on a more general mesh. Importantly, and as evidenced in Fig. 7, this result suggests that with enough grid points and/or computational power, we can reproduce the same types of solutions as that obtained with FFT or trapezoidal methods, regardless of the underlying mesh. Moreover, early experiments suggest that deploying higher-order polynomial approximations in our collocation scheme enables us to calculate the integral in (6) more accurately without such dramatic increases in mesh size, thus potentially circumventing the need for significant increases in computational power.

### Torus

In this section we deploy the MMP algorithm in order to solve the NFM in (2) on the curved surface of a torus using both linear collocation and the trapezoidal rule for comparison. Note that for any given triangulation of the torus, the collocation techniques described in Section 2 can be deployed directly to solve (2); however, implementation of the trapezoidal rule requires a regular (in the appropriate polar coordinate system) spatial discretisation of the torus, which is most easily obtained by considering the following parameterisation of the toroidal surface:

9 $$ (\theta, \phi) \mapsto \left( \begin{array}{c} (R+r\cos{\theta})\cos{\phi}\\(R+r\cos{\theta})\sin{\phi}\\r\sin{\theta} \end{array}\right) = \left( \begin{array}{c}x\\y\\z \end{array}\right). $$

**Fig. 8:**
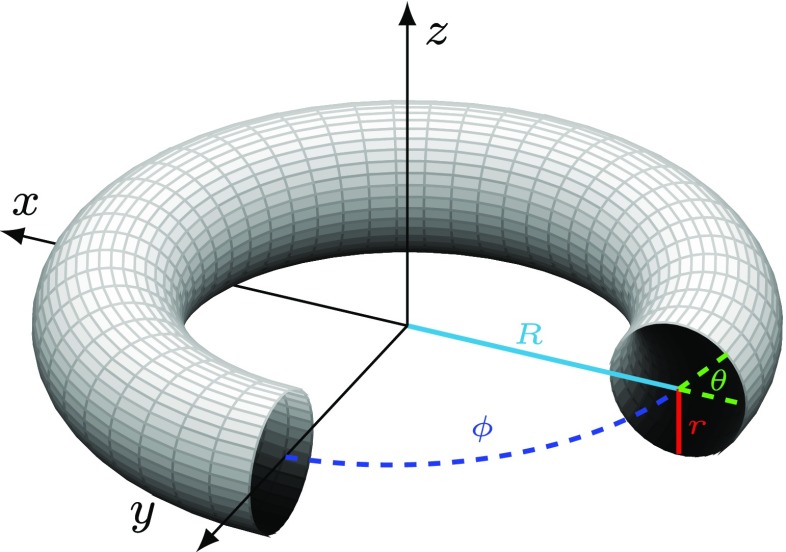
Parameterisation of a torus by coordinates (*𝜃*, *ϕ*)

The geometrical meaning of the major curvature radius *R*, the minor curvature radius *r*, and the angles *𝜃* and *ϕ* are shown in Fig. 8.

Importantly, the above parameterisation allows us to rewrite (2) as follows:

10 $$ \begin{array}{ll} \frac{\partial u(\theta,\phi)}{\partial t} &= -u(\theta,\phi)-a(\theta,\phi)+ A{\int}_{0}^{2\pi}{\int}_{0}^{2\pi} w((\theta,\phi),(\theta^{\prime},\phi^{\prime}))S(u(\theta^{\prime},\phi^{\prime})-h)r(R+r\cos{\theta^{\prime}})\mathrm{d}\theta^{\prime}\mathrm{d}\phi^{\prime},\\ \tau\frac{\partial a(\theta,\phi)}{\partial t} &= Bu(\theta,\phi)-a(\theta,\phi), \end{array} $$

which is in a form that enables us to apply the trapezoidal rule directly to solve the integral part of the equation, *i.e.*

$$\begin{array}{@{}rcl@{}} I(\theta, \phi) = &{\int}_{0}^{2\pi}{\int}_{0}^{2\pi} w((\theta,\phi), (\theta^{\prime},\phi^{\prime}))S(u(\theta^{\prime},\phi^{\prime})-h)\times \\ &r(R+r\cos{\theta^{\prime}})\mathrm{d}\theta^{\prime}\mathrm{d}\phi^{\prime}. \end{array} $$

Note that in the above, we have used the fact that the surface area element for the torus is given by

$$\mathrm{d}{\Omega}(\theta,\phi) = r(R+r\cos{\theta})\mathrm{d}\theta\mathrm{d}\phi, $$

which can easily be derived from the first fundamental form. It is also worth pointing out that the above integral is not a convolution integral and so we cannot use FFT techniques to solve (2) on a torus, or indeed on more general surfaces.

**Fig. 9:**
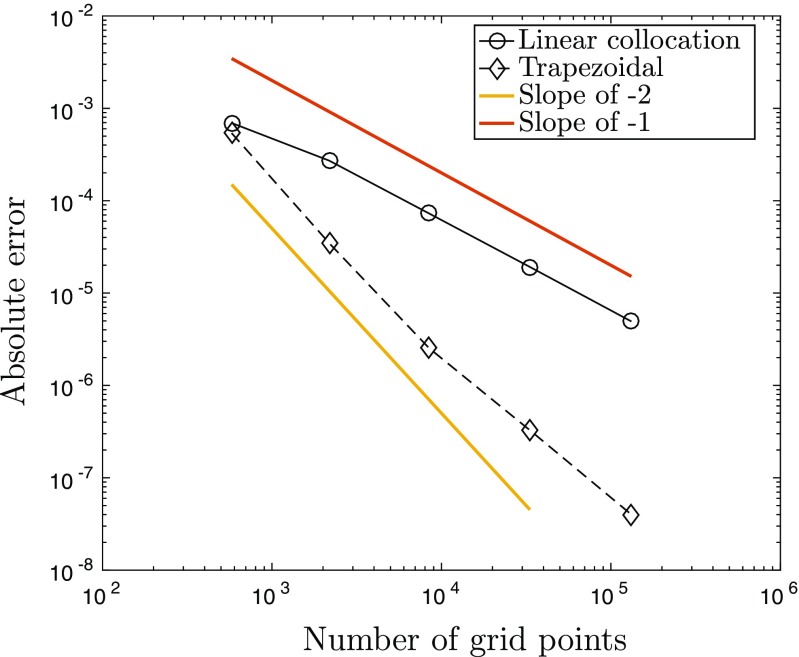
A plot of the error versus grid size when computing the integral in (2) on a regular polar coordinate grid of the torus using the trapezoidal rule (dashed line) and on a triangulation whose nodes coincide with the same grid using linear collocation (solid line)

We compared the accuracy of both linear collocation and the trapezoidal rule by considering the integral in (2) for the case when ${\Omega } = \mathbb {T}^{2}$, *i.e.* the closed surface of a torus, with minor radius *r* = 2 and major radius *R* = 4.5. As with our previous analysis, we set the unknown function $u(\theta ^{\prime }, \phi ^{\prime })= w((0,0),(\theta ^{\prime }, \phi ^{\prime }))$, that is the connectivity kernel given in (3), with the distance function *d* calculated numerically using the MMP algorithm. Starting from a regular, initial grid of *N*_0_ = 162 nodes, obtained by applying the spatial discretisation

11 $$ \begin{array}{ll} \theta_{i} &= \theta_{0}+i\delta\theta,\quad i = 0,1,\ldots, 8, \\ \phi_{j} &= \phi_{0}+j\delta\phi,\quad j = 0,1,\ldots, 17, \end{array} $$

we solved the integral on a sequence of increasingly fine meshes, in an identical manner to that described in Section 3.1. A regular triangulation was constructed from the rectangular tesselation (see, for example, Fig. 8) resulting from the aforementioned grid by setting each grid point as a vertex, and subdividing each rectangular element into two triangles. The results are displayed in Fig. 9. In particular, we see that the orders of convergence are linear for the piecewise linear collocation method and quadratic for the trapezoidal rule.

**Fig. 10:**
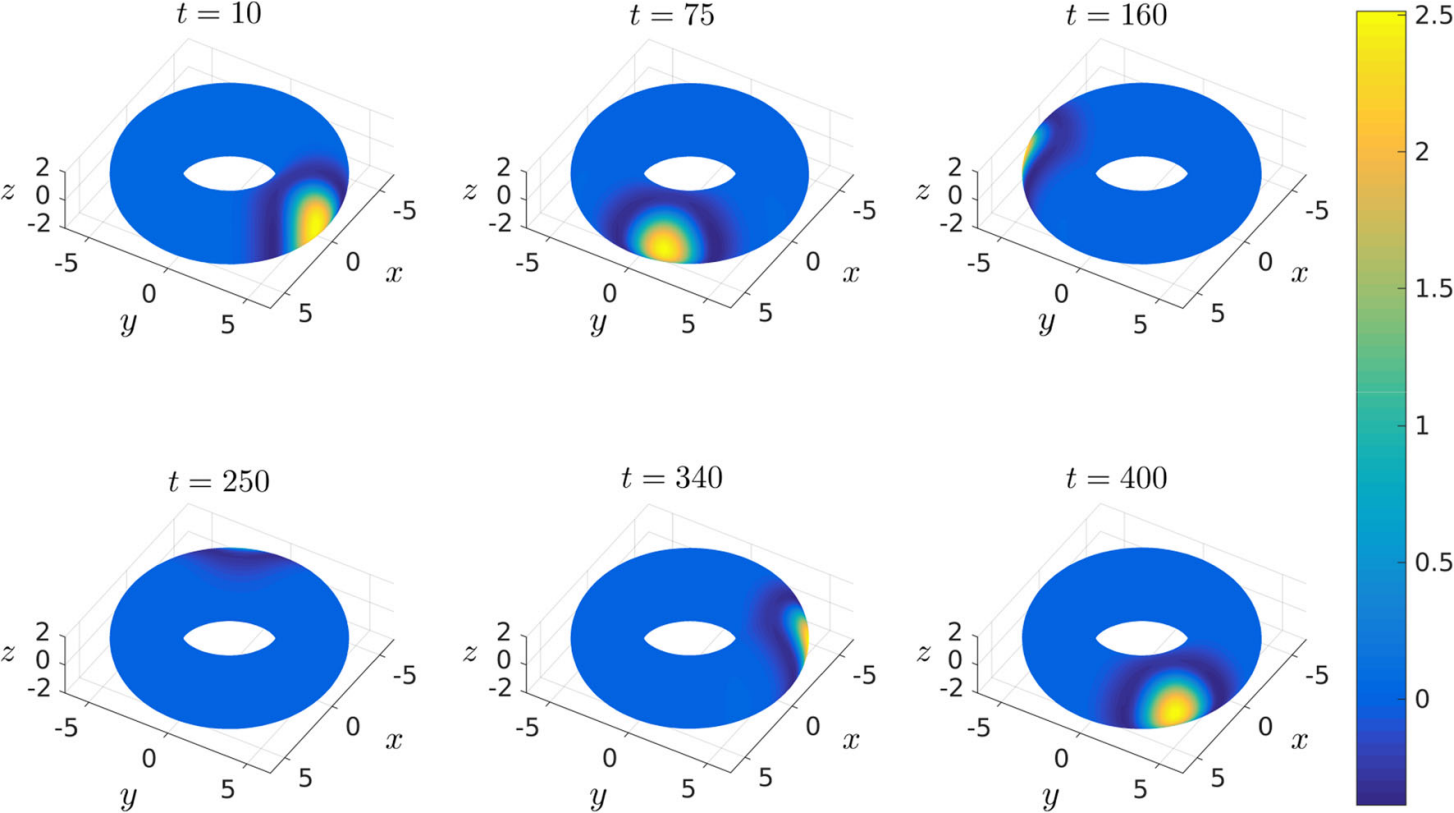
Snapshots of a travelling bump solution propagating clockwise on a torus with minor curvature radius *r* = 2.5 and major curvature radius *R* = 4.5 computed by solving equation (10) using linear collocation

We tracked the evolution of the neural activation *u*, which was initially set equal to two in a rectangular area centered on *𝜃* = *ϕ* = 0, whilst the recovery variable *a* was set equal to 1.5 in a rectangular area shifted to the right in the direction of the azimuthal angle *ϕ*, relative to the initial stimulus *u*. Equation (2) was first solved on a regular triangulation as described above. The ODEs resulting from this spatial discretisation (see (5) in the case of linear collocation) were then solved for *T* = 400 using the built-in MATLAB routine ode45, with absolute and relative tolerences both set to 1e − 6. Figure 10 shows a stable travelling bump solution of (2) propagating clockwise on the outside of the torus (*i.e.𝜃* = 0), computed by solving (2) using collocation on a regular grid of *n*_*v*_ = 8256 nodes. Note that similar results were obtained using the trapezoidal method.

**Fig. 11:**
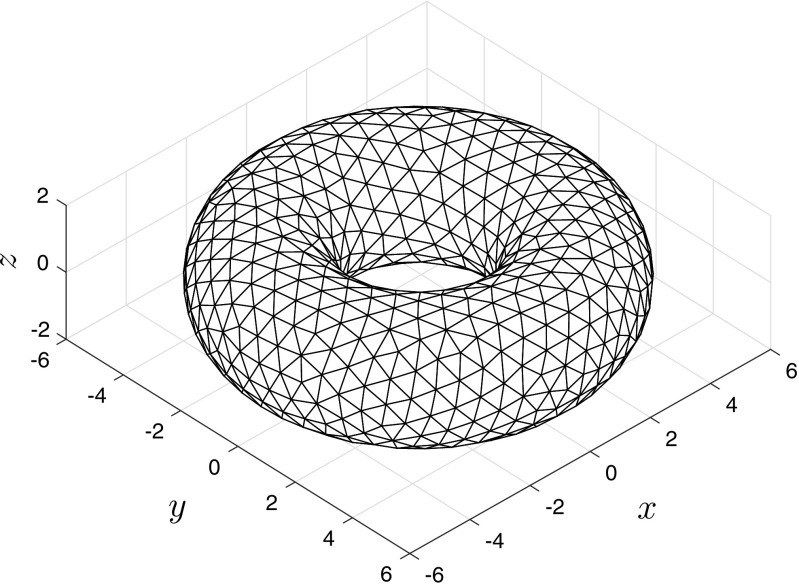
Illustration of a general triangulation of the torus generated using the DistMesh package (Persson and Strang 2004)

In addition to the above experiments, we also solved (2) on a general triangulation of the torus with *n*_*v*_ = 11094 nodes, obtained using the DistMesh package – an illustration of which is shown in Fig. 11. Importantly, we were able to reproduce the travelling bump solution displayed in Fig. 10 on this more general mesh using linear collocation. Recall that the standard product trapezoidal rule deployed in our investigations requires a domain formed via a tensor product of intervals and so is not applicable in this more general case. We note, however, that it is possible to devise quadrature rules (including the trapezoidal scheme) on general triangulated domains, but this lies outside the scope of the current work (the interested reader should see, for example, Rathsfeld 2000, Carstairs and Miller^2017^).

We note that to attain an accurate solution of (2) on an unstructured mesh using planar-triangle elements requires a finer discretisation (an increase of some 35% in the number of nodes needed to solve (2) on an unstructured as opposed to structured mesh), thus resulting in associated increases in computational resources. However, preliminary investigations suggest that considerable gains in computation can be made by increasing the order of the basis functions used (as suggested in the planar case in Section 3.1) in tandem with a more accurate representation of the geometry of the problem by deploying curved elements (Bardhan et al. 2007).

**Fig. 12:**
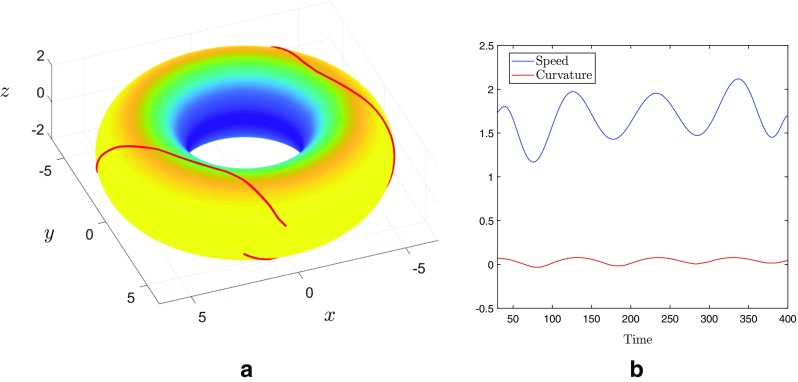
**a** The tracked path of a bump solution of (2) following a non-geodesic trajectory on the torus. Toroidal regions of maximum (positive) curvature are coloured yellow while regions of minimum (negative) curvature are coloured blue. **b** The curvature (red line) and speed (blue line) of the bump solution along the trajectroy shown in (**a**)

The travelling bump solutions considered in this paper propagate at constant speed along the geodesic curve given by the outer equator of the torus, and perhaps more importantly along trajectories of constant curvature in the direction of travel. However, we have also considered travelling bump solutions that propagate along non-geodesic trajectories, by considering different initial choices of the recovery variable, *a*. Figure 12a shows the path of such a solution as it traverses the torus. In particular, we find that solutions following non-geodesic paths travel with spatially variable speed. Moreover, the region of greatest negative curvature along the inner equator acts as a barrier, in the sense that solutions travelling along non-geodesic paths are unable to pass through this region, and instead we observe oscillatory-like behaviour as the bump solution repeatedly crosses the outer equator. This behaviour is further evidenced in Fig. 12b, in which we plot both the speed of the bump solution, as well as the Gaussian curvature as functions of the position along the trajectory plotted in Fig. 12a. We have also considered solutions passing through so-called meridian geodesics, *i.e.* paths of fixed azimuthal angle, and found that such bump solutions travel at constant speed and pass through the inner equator unhindered (results not shown).

### Cortical surface of a rat

**Fig. 13:**
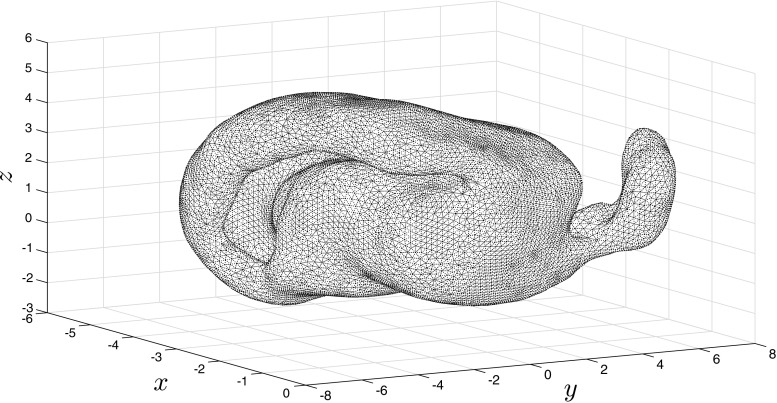
A triangulation of the left hemisphere of the rat cortex

Spatial coordinates for the cortical surface of the rat were obtained via the CARET software package (Van Essen 2012) and processed using the CARET MATLAB toolbox. Restricting to the left hemisphere, we deploy the triangulation of the rat cortex provided by the CARET software, with nodes positioned on the *n*_*v*_ = 9623 available data points (see Fig. 13). We tracked the evolution of neural activity *u*, which was initially set equal to two in a small region (1% of the total nodes in the mesh) surrounding a node selected at random, whilst the recovery variable *a* was set equal to 1.5 in an equivalently sized, partially overlapping region of nodes. Note that the initial position of the recovery variable determines the direction of propagation and so we repeated this process a number of times in order to gain insight into how both the geometry, as well as the site, and form, of activity initiation, influences propagation travelling patterns of the localised bump solutions admitted by (2). As with our earlier experiments, the ODEs in (5) were solved for *T* = 400 using the built-in MATLAB routine ode45, with absolute and relative tolerences both set to 1e − 6.

**Fig. 14:**
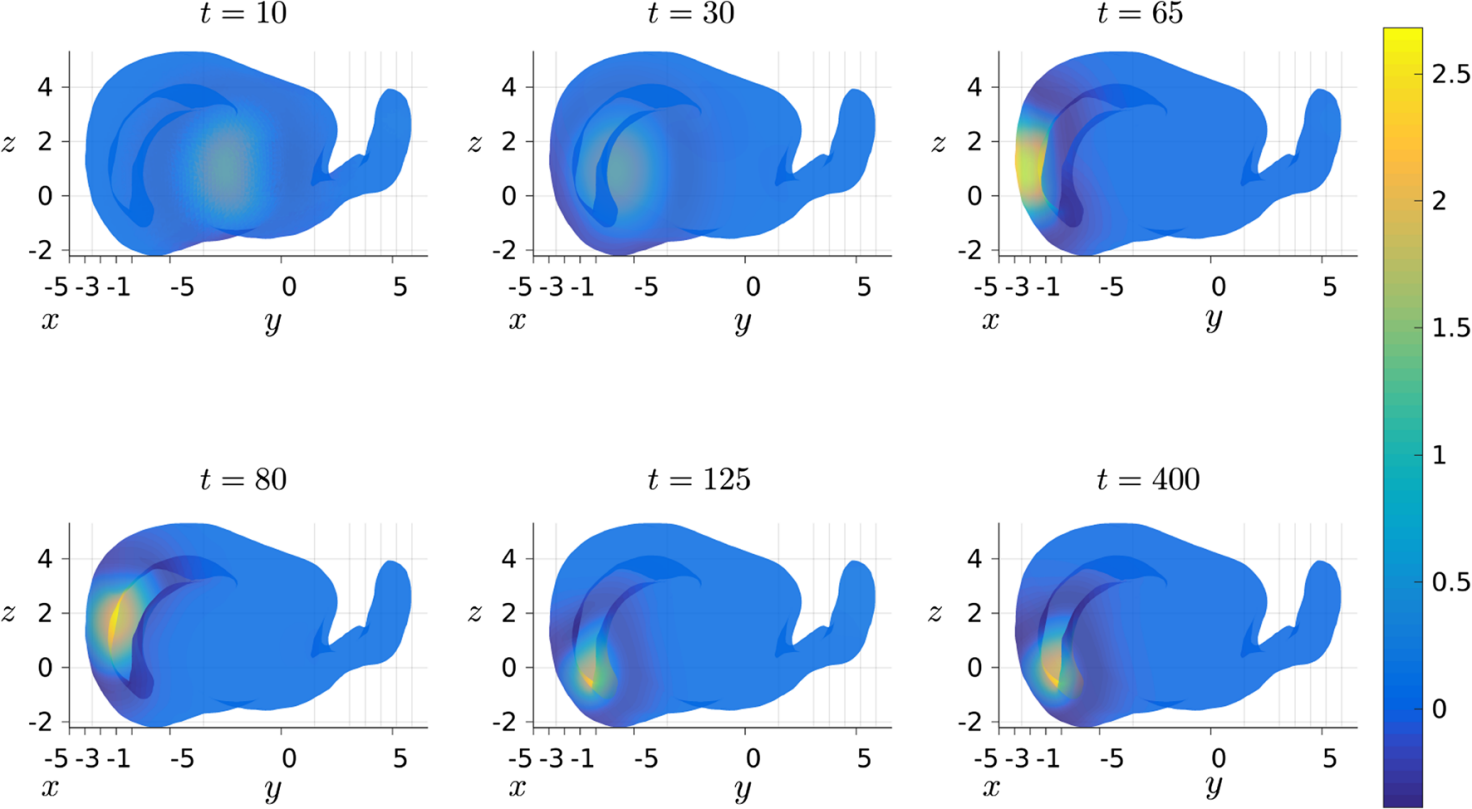
Snapshots of bump solutions of (2) propagating on the surface of the rat cortex

As an illustration, Fig. 14 shows the progression of a typical stable bump solution of (2), for several selected time points. Importantly, we find regardless of the initial direction of propagation that solutions tend to one of two steady states: either they settle on the large folded region on the underside of the rat brain (see the panel in bottom right corner of Fig. 14), or they get stuck in the transition between the main body of the brain and the tail-like structure to the rear – see Figure S1 in the supplementary material for an example of such a solution. We remark that unlike the solutions obtained on the torus, *all* solutions obtained for the rat brain traverse regions of both positive and negative Gaussian curvature, and as a result of this variation in curvature, *all* observed solutions travelled along non-geodesic trajectories with spatially-variable speed. Crucially, this is in direct contrast to solutions obtained on the flat, periodic square, as well those solutions on the torus following geodesic trajectories of constant curvature, both of which travelled at constant speed. Note that preliminary studies in this direction suggest that both the propagation path and the variation in speed of these solutions is largely explained by the gradient in the Gaussian curvature of the surface under consideration. These matters shall be further investigated in a follow-on manuscript that is currently under preparation.

## Discussion

In this paper, we have presented a computational technique for solving neural field models (NFM) on curved geometries and investigated the influence of the underlying mesh on these solutions. More specifically, we compared numerical simulations of the propagation of neural activity within three different geometries: a flat periodic square, the surface of a torus and the cortical surface of the rat brain. Note that to the best of our knowledge this is the first time that a NFM such as (1) has been solved on a curved geometry for which no analytic formulae for geodesic distance exists. Importantly, in the case of the periodic square, we found that collocation techniques are capable of replicating travelling bump solutions found using more standard techniques, such as Fourier based methods or the trapezoidal rule, using general, non-Cartesian meshes, more akin to the types of meshes derived from modern neuroimaging studies. This result, coupled with efficient numerical techniques for computing geodesic distances on triangulated surfaces, allows us to extend these algorithms with confidence to determine solutions of neural field models on curved geometries, such as the torus and rat brain considered herein.

A key feature of this work is that we deploy neuroimaging data from the left hemisphere of the rat brain, alongside efficient numerical procedures for computing geodesic distances, in order to study the behaviour of localised spot-like solutions of a non-local neural field model. Importantly, preliminary results suggest that cortical geometry influences profoundly both the propagation speed and path of such localised bump solutions, thus leading us to conclude that studies that do not account for the folded structure of the cortex risk simplifying neural activation dynamics in a potentially significant way. Note that we limit the current study on comparisons of activity propagation on the brain surface to short-range connections between, say, cortical columns; however, our approach is capable of including long-range white matter connections via the choice of a suitable, possibly experimentally defined, connectivity kernel. Indeed, incorporating macro-scale (white matter) connectivity within the NFM, thus more accurately reflecting neural mechanisms of relevance to bumps, waves and more general patterns of neural activity in the brain, is an important area of future research.

A number of recent studies (Jirsa et al. 2001; Kroos et al. 2016) have investigated the relations of cortical geometry to the nucleation and propagation of waves. However, such studies typically rely on special choices of the connectivity kernel in order to obtain a PDE formulation of the NFM, thus ignoring important physiological details, such as the role of cortical inhibition, an important and well-known mechanism for controlling the propagation of neural activity. Although such studies have reported significant differences in wave propagation properties due to geometric effects, the ommission of key inhibitory mechanisms means that features of relevance to both healthy and pathological spreading of neural activity are potentially missed. One way to test this, would be to repeat the analysis of Jirsa et al. (2001), in which the authors attempt to replicate observed EEG/MEG activity patterns on a human cortical surface using a NFM with a homogeneous connectivity function, to see whether we can better recreate observed neural activity by deploying the full integral model, including both short-range excitatory as well as longer range inhibitory connections. In this way we would hope to highlight the influence of cortical inhibition on propagation dynamics, in isolation.

To conclude, our work is significant for a number of reasons. Firstly, we introduce a novel numerical procedure for integrating NFMs on arbitrary two-dimensional surfaces, thus opening up the possibility of studying more physiologcally realistic systems, including, for example, accurate cortical geometries and/or connectivity kernels displaying regional heterogeneity. And secondly, preliminary results on the curved surface of the rat brain suggest that Gaussian curvature has a significant impact on both the speed and path of propagating neural activity, and so an important open question is to determine to what extent the folded structure of the cortex influences mechanisms describing the interaction between complex wave dynamics and the observed frequencies of emergent brain rhythms. Future work shall focus on determining the influence of curvature upon the nucleation and propagation of the spatially localised bump-like solutions observed in this study, using numerical bifurcation techniques, as well as considering more complicated NFMs that include, for example, time delays, or more general connectivity kernels that incorporate experimental data thus better reflecting brain physiology.

## Electronic supplementary material

Below is the link to the electronic supplementary material.

(PDF 1.88 MB)
