# Supplementary material for: A numerical simulation of neural fields on curved geometries
Source: J Comput Neurosci. 2018 Oct 11;45(2):133–45. doi: 10.1007/s10827-018-0697-5 (PMC6208890; doi:10.1007/s10827-018-0697-5)
Supplement: Supplementary file 1 — (PDF 1.88 MB) [file 10827_2018_697_MOESM1_ESM.pdf]

Supplementary material: A numerical simulation of neural fields on curved geometries

Steady state solutions of the NFM (2) when studied on the rat cortex

As mentioned briefly in the main manuscript, when considering travelling bump solutions of Equation (2) on the rat cortex we obtain two different steady states, regardless of the initial condition. Solutions either settle on the large folded region on the underside of the rat brain (as shown in Figure 14 in the main manuscript); or they get stuck in the transition between the main body of the brain and the tail-like structure to the rear. Figures S1 and S2 show such a solution viewed from both the front of and the back of the rat brain. An interesting point here is that this solution converges not to a bump solution, but to a ring solution about the tail - this behaviour is the subject of ongoing study and will be further described elsewhere.

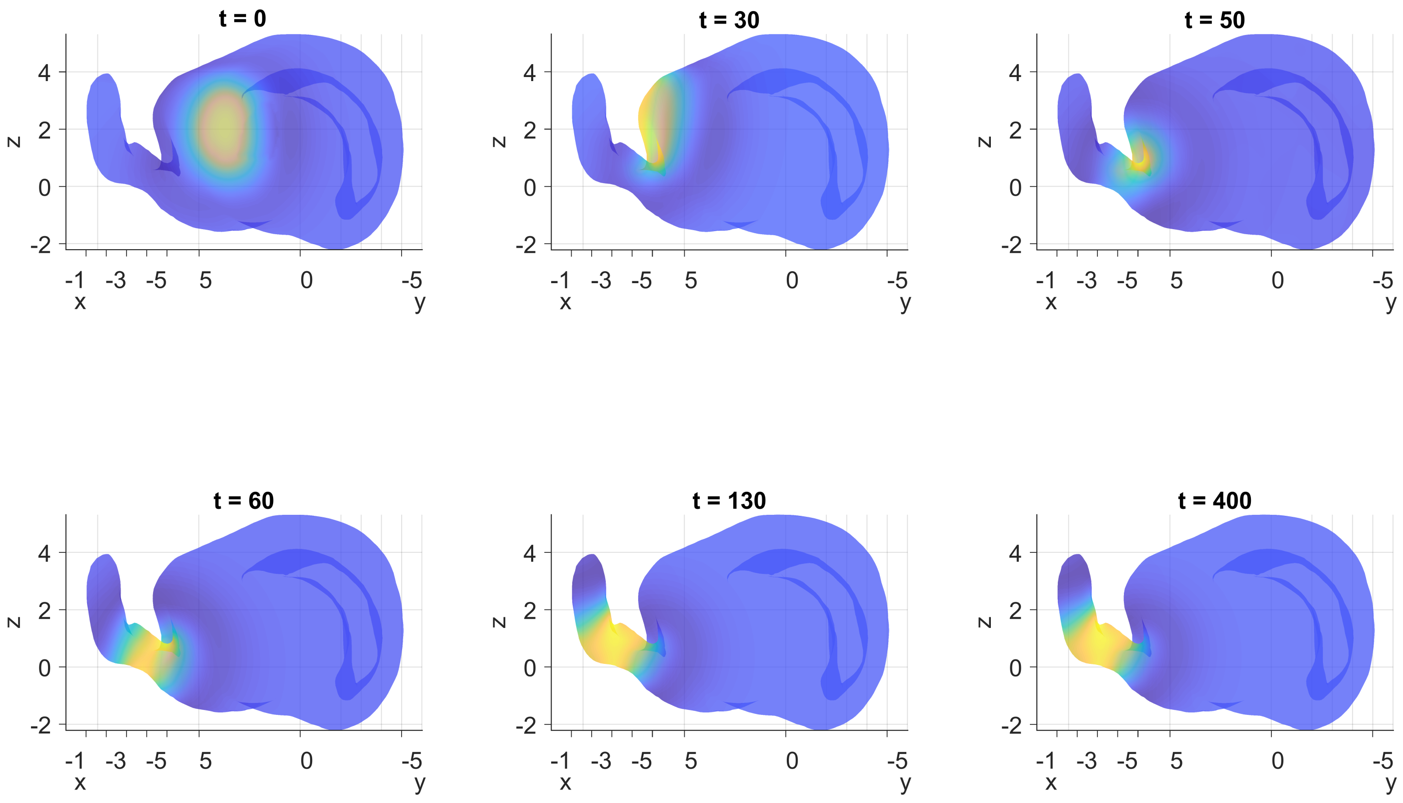

Figure S1: Snapshots of bump solutions of (2) from the main manuscript propogating on the surface of the rat cortex. (front view)

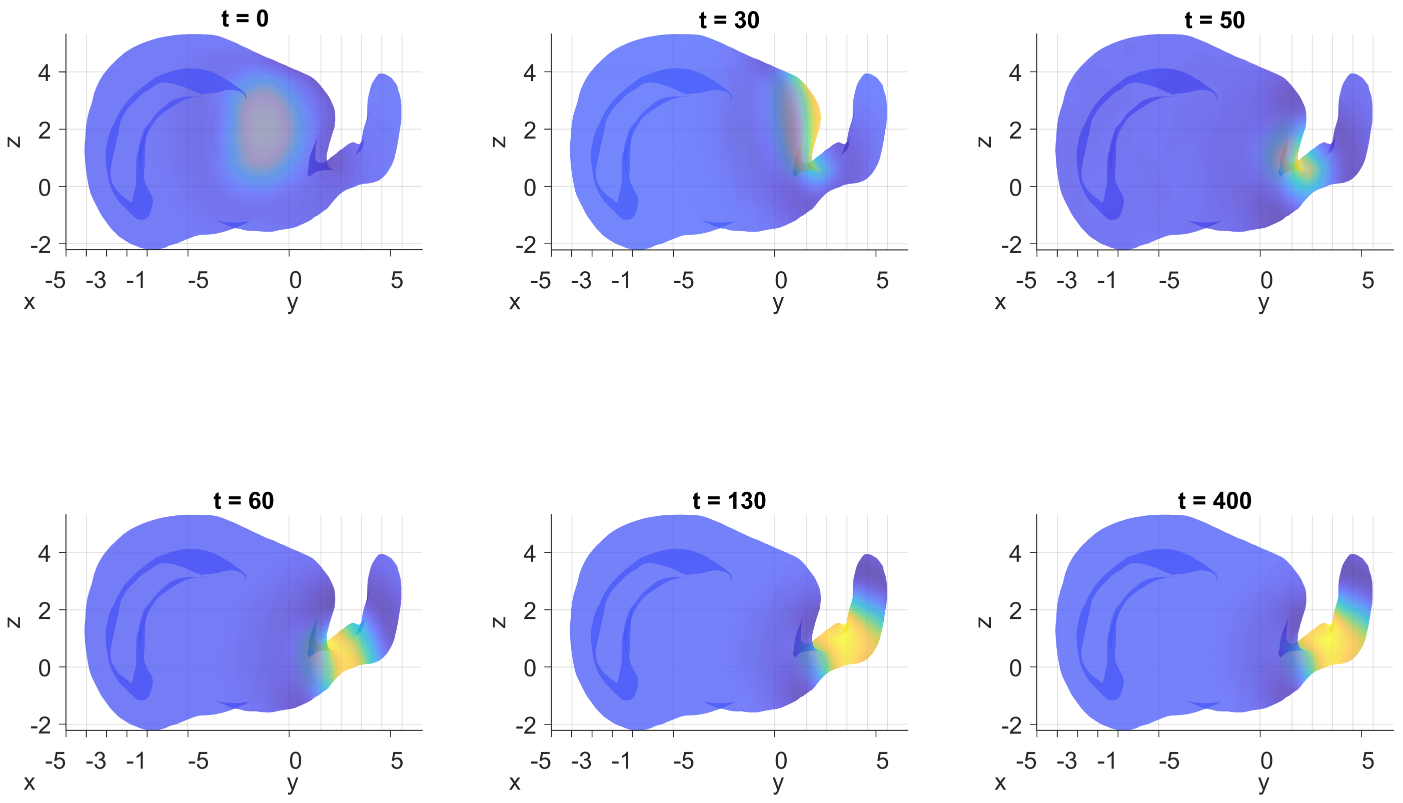

Figure S2: Snapshots of bump solutions of (2) from the main manuscript propogating on the surface of the rat cortex. (rear view)
